# Artificial Intelligence and Machine Learning-based prediction of tuberculosis treatment failure: A systematic review and meta-analysis

**DOI:** 10.1371/journal.pone.0355124

**Published:** 2026-08-17

**Authors:** Rogers Kamulegeya, Rose Nabatanzi, Derrick Semugenze, Faridah Mugala, Mercy Takuwa, Emmanuel Nasinghe, Denis Musinguzi, Sharon Namiiro, Andrew Katumba, Willy Ssengooba, Joyce Nakatumba-Nabende, Florence Nameere Kivunike, David Patrick Kateete

**Affiliations:** 1 Department of Immunology and Molecular Biology, Makerere University College of Health Sciences (MakCHS), Kampala, Uganda; 2 Department of Computer Science, College of Computing and Information Sciences, Makerere University (CoCIS), Kampala, Uganda; 3 Department of Information Technology, College of Computing and Information Sciences, Makerere University (CoCIS), Kampala, Uganda; 4 Masaka Regional Referral Hospital, Masaka, Uganda; 5 Makerere University Lung Institute (MLI), Kampala, Uganda; 6 Department of Electrical and Computer Engineering, College of Engineering, Art and Design, Makerere University (CEDAT), Kampala, Uganda; 7 Department of Global Health and Amsterdam Institute for Global Health and Development, Amsterdam University Medical Centers, Amsterdam, Netherlands; Gulu University, UGANDA

## Abstract

**Background:**

Tuberculosis (TB) remains a leading cause of infectious disease mortality worldwide, and treatment failure contributes to ongoing transmission, drug resistance, and poor clinical outcomes. Artificial intelligence (AI) and machine learning (ML) approaches have attracted growing interest in predicting TB treatment outcomes, but the literature is heterogeneous and lacks a comprehensive synthesis.

**Methods:**

We systematically searched PubMed/MEDLINE and Embase (January 2000–October 2025) for studies developing or validating AI/ML models to predict TB treatment failure. Two reviewers independently screened records and extracted data on study characteristics, predictor modalities, algorithms, validation strategies, and performance metrics, i.e., area under the curve (AUC), sensitivity, specificity, and confidence intervals. Studies reporting AUC with confidence intervals or sufficient data for calculation were included in random-effects meta-analysis. Missing standard errors were estimated from sample sizes and event rates using established methods. Risk of bias was assessed using PROBAST. Subgroup analyses and meta-regression explored heterogeneity, and publication bias was assessed using funnel plots, Egger’s test, and trim-and-fill analysis. The study is registered with PROSPERO (CRD420251101443).

**Results:**

Thirty-four studies met the inclusion criteria. Publications increased markedly from 2019 onwards (91% of studies). Tree-based methods predominated (52.9%), and multimodal models (≥3 data types) were used in 41.2% of the studies. Nineteen studies (100,790 participants) contributed to the meta-analysis. The pooled AUC was 0.836 (95% CI 0.799–0.868), with substantial heterogeneity (I² = 97.9%). In subgroup analyses, studies including HIV-positive participants showed lower discrimination (AUC 0.748) than those excluding them (0.924). Only eight studies (23.5%) performed external validation, and only one study (2.9%) was rated low risk of bias overall (PROBAST), primarily due to analytical domain deficiencies. Egger’s test suggested publication bias (p = 0.024). Major evidence gaps included underrepresentation of high-burden countries, HIV-affected populations, social determinants, pediatric TB, and extrapulmonary disease.

**Conclusions:**

AI/ML models for predicting TB treatment failure show promising discrimination but are not yet ready for routine clinical implementation. Performance varies substantially across populations and settings, and methodological limitations, including inadequate validation, poor calibration assessment, and high risk of bias, limit confidence in current estimates. Future research should prioritize rigorous external validation, calibration assessment, and development in underrepresented populations, particularly HIV-affected and high TB burden settings.

## Introduction

Tuberculosis (TB), caused by *Mycobacterium tuberculosis*, is a leading cause of death worldwide, with an estimated 10.8 million incident cases and 1.25 million deaths as in 2023 [1]. While standard antibiotic regimens are generally curative, treatment failure remains a clinical and programmatic challenge. Such failures lead to persistent infectiousness, prolonged morbidity, relapse, amplified drug resistance, and continued community transmission [2–7].

The risk of unsuccessful treatment is shaped by interacting pathogen, host, health system, and socio-structural determinants. Pathogen characteristics like drug resistance [6,7] interact with host vulnerabilities such as HIV coinfection, diabetes, and malnutrition [8,9]. Simultaneously, systemic barriers; including poverty, stigma, and poor treatment access; further undermine adherence and outcomes [5,10–13]. Because these multidimensional factors rarely operate in isolation, traditional statistical models relying on limited clinical variables often struggle to accurately predict individual patient risk [14–18]. Consequently, artificial intelligence (AI) and machine learning (ML) have emerged as promising alternatives capable of integrating high-dimensional, multi-modal data (e.g., clinical, genomic, socioeconomic) to generate personalized prognostic estimates [9–12,14–18].

Despite growing application of AL and ML approaches to predict treatment failure and other poor TB treatment outcomes [19–26], existing literature remains highly fragmented. Studies vary widely in target populations, ranging from drug-susceptible to multidrug-resistant and extensively drug-resistant TB [27]; in epidemiologic context, including high TB burden, high TB and HIV burdened, and lower TB burden settings; in predictor variables; in model architectures; in validation strategies; and in the outcomes and performance metrics reported [21,22,28,29]. Crucially, it remains unclear how well models developed in data-rich or low-burden contexts generalize to high-burden or low-resource settings, where baseline risks, HIV coinfection rates, and data availability differ substantially [30–33]. It is also unknown whether routinely unavailable inputs, such as genomic sequencing data or complex pharmacokinetic measures, provide sufficient incremental value to justify their cost in these environments. These differences make it difficult to determine overall predictive performance, identify the most informative data modalities, and judge whether current models are sufficiently robust for clinical translation.

Current prognostic models for TB inadequately capture complex, multidimensional patient risks. While AI and ML offer robust frameworks to overcome these limitations, their clinical translation necessitates rigorous evaluation. To address these evidence gaps, we conducted a systematic review and meta-analysis evaluating the predictive performance of AI/ML models for TB treatment failure. Our objectives were to compare model efficacy across diverse epidemiological and socioeconomic contexts, characterize prevalent predictor domains and data modalities, and assess methodological quality and risk of bias using PROBAST. By evaluating validation rigor and generalizability, we aim to identify critical knowledge gaps to inform the future development and equitable clinical implementation of these predictive tools [34–36].

## Methods

### Study design and registration

This systematic review and meta-analysis were conducted in accordance with the Preferred Reporting Items for Systematic Reviews and Meta-Analyses (PRISMA) 2020 statement and informed by methodological guidance for systematic reviews of prediction model studies and diagnostic accuracy evidence [37,38]. The protocol was prospectively registered in PROSPERO (CRD420251101443).

### Search strategy

We systematically searched PubMed/MEDLINE and Embase for studies published from 1 January 2000–31 October 2025. The search combined controlled vocabulary terms, including Medical Subject Headings and Emtree headings, with free-text terms related to tuberculosis, artificial intelligence, machine learning, prediction modeling, and treatment outcomes. Search development was informed by established systematic review principles and structured to maximize sensitivity and reproducibility. The full search strategy is provided in the S5 File.

### Eligibility criteria

Eligibility was defined using a modified Population, Intervention, Comparator, Outcome framework. We included studies of adults or children with active pulmonary or extrapulmonary TB, including both drug-susceptible and drug-resistant disease, who were undergoing anti-TB treatment. Eligible studies developed, validated, or implemented an AI or ML model for prediction of TB treatment failure or a closely related poor treatment outcome. Because this was a review of prediction modeling studies, a conventional comparator was not required.

Studies were required to report at least one quantitative measure of predictive performance, such as area under the curve (AUC), sensitivity, specificity, accuracy, F1-score, calibration, or another validation metric.

We excluded studies that used only conventional statistical methods without an AI/ML component; studies focused exclusively on TB diagnosis, latent TB infection, or transmission without treatment outcome prediction; studies without original data, including reviews, editorials, and commentaries; animal or in vitro studies; and non-English publications.

Pediatric studies were eligible because the review aimed to capture the full scope of published prediction modeling for TB treatment outcomes. However, age-specific subgroup analysis was planned only if sufficient pediatric studies were available; because eligible pediatric evidence was sparse and inconsistently reported, pediatric studies were synthesized descriptively, and the implications of age-related heterogeneity are addressed in the Discussion and Limitations.

### Study selection

Three reviewers independently screened titles and abstracts using Rayyan. Full texts of potentially eligible records were then reviewed independently by the same reviewers against the predefined criteria. Disagreements were resolved through discussion or, when necessary, consultation with a fourth reviewer. The study selection process was documented using a PRISMA flow diagram.

### Data extraction

A standardized extraction form was developed a priori and piloted before full extraction, consistent with guidance for prediction model reviews [38,39]. Extracted data included study design, setting, country, recruitment source, study period, sample size, population characteristics, drug susceptibility profile, HIV status, comorbidities, treatment failure definition, follow-up duration, predictor domains, data modalities, feature engineering and selection methods, algorithms evaluated, model validation strategies, hyperparameter tuning, discrimination measures (AUC with 95% confidence intervals, sensitivity, specificity, positive predictive value, negative predictive value, F1‑score) and calibration metrics (calibration slope, calibration‑in‑the‑large, calibration plots), explainability methods, and data required for Prediction model Risk Of Bias Assessment Tool assessment [40–43].

For the quantitative synthesis, two reviewers independently extracted performance metrics, specifically focusing on the C-statistic/AUC with corresponding 95% confidence intervals (CIs). Where available, we extracted raw confusion matrix data (true positives, false positives, true negatives, false negatives) and the total sample size for the outcome of interest to facilitate risk variance calculations. When several models were reported in one study, we extracted the best-performing or primary model according to the authors’ stated criterion, while recording alternative models descriptively. Studies entered the AUC meta-analysis only when an AUC and sufficient information to estimate uncertainty were available. When confidence intervals were reported, standard errors were calculated from the interval width on the logit-transformed AUC scale. When confidence intervals or standard errors were unavailable and could not be reliably estimated, the study was retained in the systematic review but excluded from quantitative pooling.

### Risk of bias assessment

Methodological quality and risk of bias were assessed using the Prediction model Risk Of Bias Assessment Tool, which evaluates prediction studies across the domains of participants, predictors, outcome, and analysis [28,29]. Two reviewers independently conducted PROBAST assessments. Each domain was rated as low, high, or unclear risk of bias alongside an assessment of concerns regarding applicability. An overall risk of bias rating was derived as follows: low risk if all domains were low, high risk if any domain was high, and unclear risk if information was insufficient. Disagreements between reviewers were resolved through discussion or, when necessary, consultation with a third reviewer. Inter-rater agreement was assessed using Cohen’s kappa (κ = 0.87, indicating almost perfect agreement).

### Data synthesis and statistical analysis

The primary quantitative outcome was model discrimination, summarized using the AUC. Given the inherent clinical and methodological heterogeneity in AI/ML prediction models (arising from diverse algorithms, hyperparameter tuning, and predictor sets), we utilized a random-effects modeling framework. While pooling heterogeneous models presents challenges, it provides a valuable macro-level summary of current AI/ML predictive capabilities, provided that the heterogeneity is subsequently explored through robust meta-regression and subgroup analyses.

Because the AUC values are bounded between 0 and 1, we applied a logit transformation before meta-analysis to improve statistical behavior and stabilize variance [30,38]. Standard errors were derived from reported confidence intervals when available or estimated using established methods when necessary [30]. Random-effects meta-analysis was performed using restricted maximum likelihood estimation, and pooled estimates were back-transformed to the original area under the curve scale for interpretation [44,45]. Statistical heterogeneity was assessed using Cochran’s Q and the I² statistic [46,47].

To explicitly address and explore the anticipated heterogeneity, we conducted prespecified subgroup analyses. These included stratification by tuberculosis susceptibility profile, country income context (low- and middle-income vs. high-income), and validation status (internal vs. external). Pediatric and adult populations present with distinct clinical trajectories; therefore, we prespecified an approach to analyze pediatric studies as a distinct subgroup to prevent confounding the adult model performance estimates.

Meta-regression using mixed-effects models was performed to examine whether study-level covariates such as publication year, sample size, tuberculosis burden setting, external validation, HIV inclusion, and model complexity, were significantly associated with AUC [38]. Sensitivity analyses included leave-one-out meta-analysis and restriction to studies exhibiting a low risk of bias. Publication bias was assessed using funnel plot inspection and Egger’s regression test; where asymmetry was detected, the trim-and-fill method was applied to estimate its potential effect on pooled discrimination [48–51].

All analyses were conducted in R version 4.5.2 using the meta, metafor, mada, robvis, and ggplot2 packages [45]. Statistical tests were two-sided, and a p-value of <0.05 was considered statistically significant.

## Results

### Study selection

**Fig 1:**
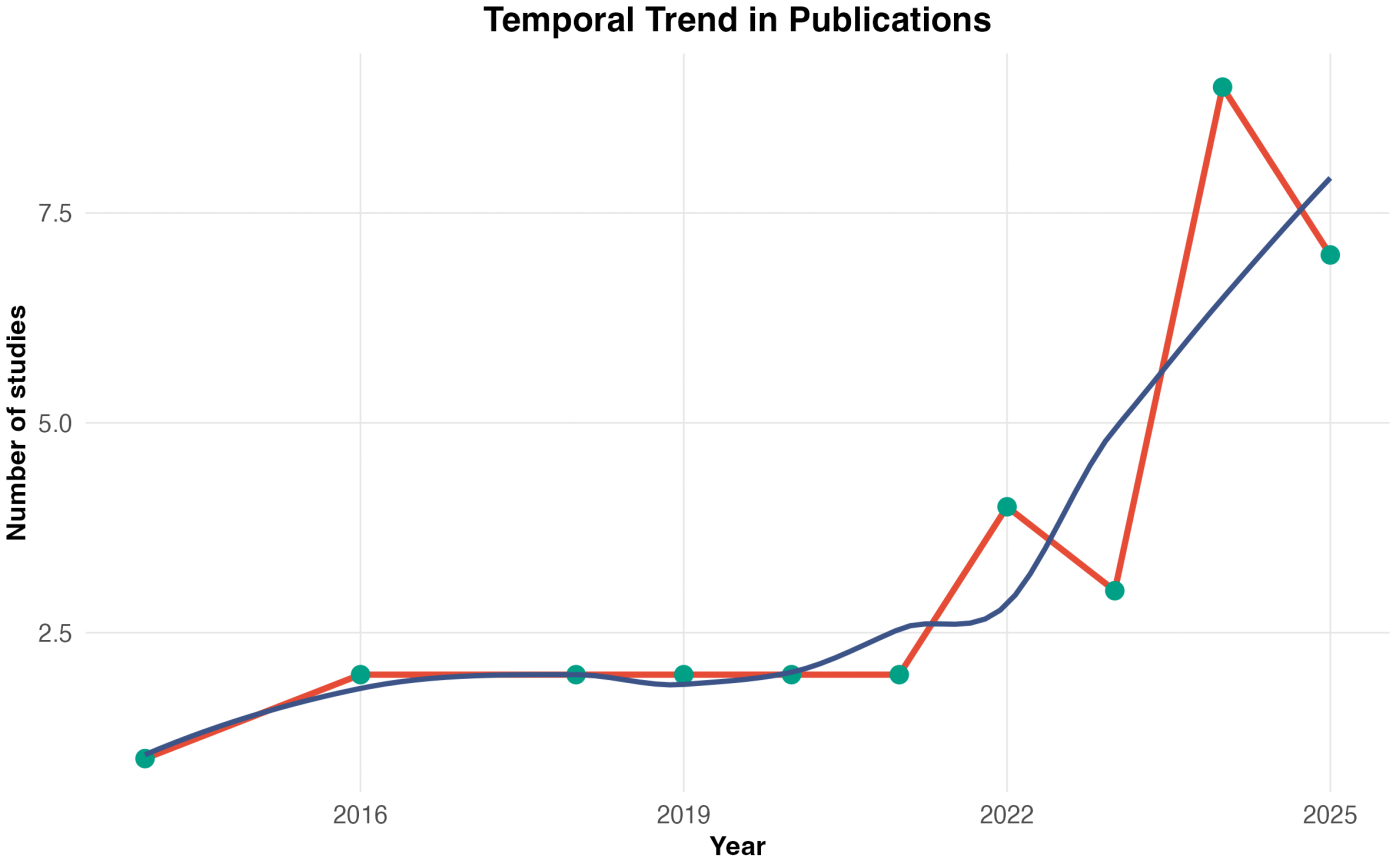
Temporal Trend in Publications. Bar chart showing the annual number of included studies published between 2014 and 2025. The number of publications remained low (1–2 per year) until 2020, then increased markedly, with 6 studies published in 2024 and 7 studies in 2025. This trend indicates a rapidly growing research interest in AI/ML applications for TB treatment outcome prediction.

The systematic search identified 1,672 records. After applying exclusion criteria and removing duplicates, 317 abstracts were screened, and 52 full-text articles were assessed. Thirty-four studies met the inclusion criteria for the systematic review, of which 19 reported area under the receiver operating characteristic curve (AUC) values with sufficient data for quantitative meta-analysis, Fig 1.

The AUC quantifies how well a model distinguishes between patients who experience treatment failure and those who do not, with values ranging from 0.5 (no discrimination beyond chance) to 1.0 (perfect discrimination).

### Study characteristics

#### Temporal and geographic distribution.

The included studies were published between 2014 and 2025, with a marked increase in recent years. Only three studies appeared before 2019, whereas 31 studies (91.2%) were published from 2019 onward, reflecting rapidly growing interest in AI and ML approaches for predicting TB treatment outcomes (Fig 1).

**Fig 2:**
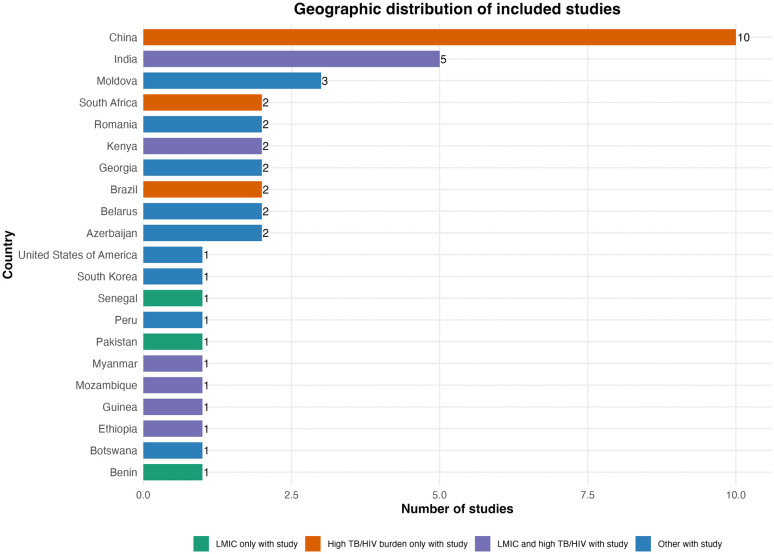
Geographic Distribution by Country Classification. Bar chart showing the distribution of included studies across countries, categorized by low- and middle-income country status and high TB/HIV burden status. China contributed the largest number of studies (n = 10), followed by India (n = 4). Many countries with high TB or TB/HIV burden contributed only one study each (e.g., Ethiopia, Kenya, Mozambique, South Africa), while several high-burden countries had no eligible studies. The “Other” category includes countries such as the United States, South Korea, and others not classified as low- and middle-income or high TB/HIV burden.

Geographically, studies were conducted across 22 countries spanning Asia, Africa, Europe, and the Americas (Table 1; Fig 2), but the distribution was uneven. China contributed the largest number of studies (n = 10, 29.4%), followed by India (n = 5, 14.7%), while multinational collaborations accounted for four studies (11.8%). Several countries contributed only one study.

**Table 1:** Geographic distribution of included studies.

Continent / Region	Country	Number of Studies	Percentage
Asia	China	10	27.3%
Asia	India	5	18.2%
Multinational*	Multinational*	4	12.1%
Eastern Europe	Moldova	3	12.1%
Eastern Europe	Romania	2	9.1%
Eastern Europe	Georgia	2	9.1%
Eastern Europe	Belarus	2	9.1%
Eastern Europe	Azerbaijan	2	9.1%
Africa	South Africa	2	6.1%
Africa	Kenya	2	6.1%
South America	Brazil	2	6.1%
Other countries†	Other countries†	1 each	3.0% each

*Multinational studies used datasets spanning multiple countries.

†Countries contributing a single study include the USA, South Korea, Senegal, Peru, Pakistan, Myanmar, Mozambique, Guinea, Ethiopia, and Botswana.

**Fig 3:**
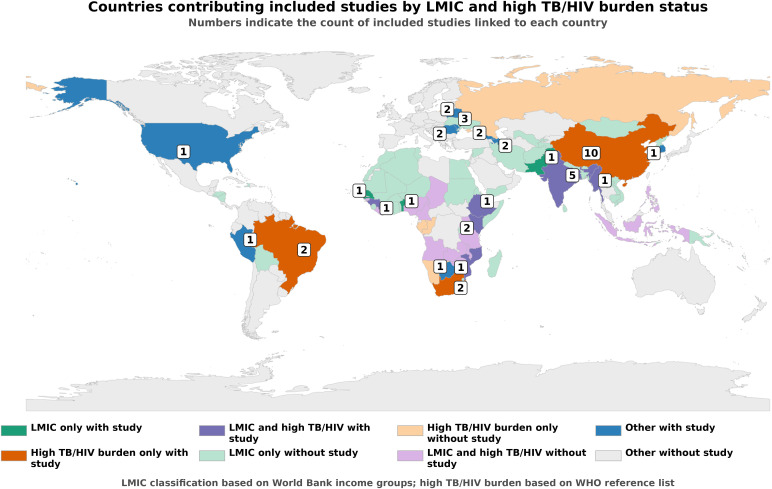
World Map of Study Distribution. World map showing the geographic distribution of included studies, with numbers indicating the count of studies linked to each country. The map highlights the concentration of studies in China and India, with sparse representation from sub-Saharan Africa, Southeast Asia, and other high-burden regions. Countries with no eligible studies are shown in light gray.

Mapping study locations against World Bank income classification and WHO high TB/HIV burden countries revealed substantial gaps. Although many studies originated from LMICs, relatively few were conducted in countries simultaneously classified as LMIC and high TB/HIV burden settings, and many high-burden countries had no eligible studies (Fig 3).

#### Study design, setting, and sample size.

Most studies used retrospective observational designs (24/34, 70.6%), while only three used prospective cohorts (Table 2). Sample sizes varied widely, ranging from 28 to 665,883 participants (median 551.5, IQR 197–4,139). Settings included tertiary referral hospitals, district and community facilities, national TB program datasets, and multicenter or international databases.

**Table 2:** Summary of study design, setting, and sample size characteristics of the included studies.

Characteristic	Category	Studies, n (%)
Study design	Retrospective cohort / retrospective observational	24 (70.6)
	Prospective cohort / prospective clinical study	3 (8.8)
	Nested case–control or case–control	3 (8.8)
	Mathematical / transmission modeling studies	2 (5.9)
	Longitudinal pilot / validation study	1 (2.9)
	Other hybrid designs	1 (2.9)
Study setting	Single-country datasets	30 (88.2)
	Multinational datasets	4 (11.8)
Sample size	Median sample size (IQR)	551.5 (197–4,139)
	Minimum sample size	28
	Maximum sample size	665,883

#### Population characteristics.

**Fig 4:**
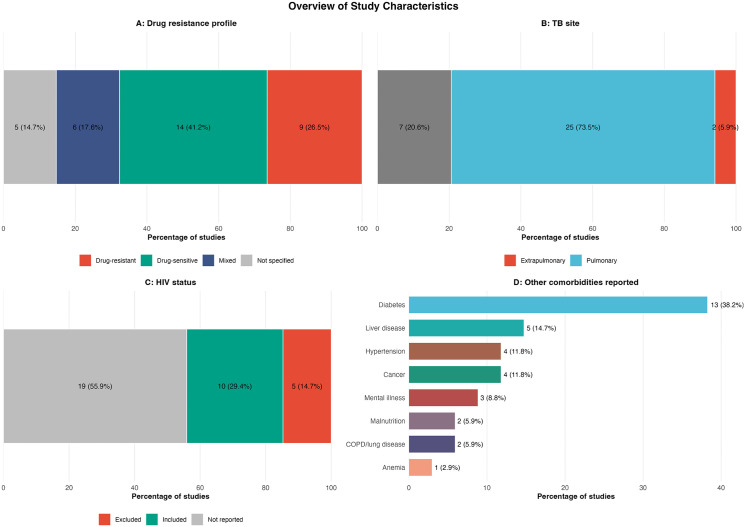
Overview of Study Population Characteristics. Four-panel figure summarizing key population characteristics of the 34 included studies. Panel A: Drug Resistance Profile. Fourteen studies (41.2%) focused on drug-susceptible TB, nine (26.5%) on drug-resistant TB, nine (26.5%) on mixed populations, and two (5.9%) did not specify resistance profile. Panel B: Tuberculosis Site. Twenty-five studies (73.5%) focused exclusively on pulmonary tuberculosis, while nine (26.5%) included both pulmonary and extrapulmonary cases. No studies focused exclusively on extrapulmonary TB. Panel C: HIV Status. Ten studies (29.4%) included HIV-positive participants, four (11.8%) explicitly excluded HIV-positive individuals, and 14 (41.2%) did not report HIV status. Panel D: Other Comorbidities Reported. Diabetes was the most frequently reported non-HIV comorbidity (13 studies, 38.2%), followed by liver disease (5, 14.7%), hypertension (4, 11.8%), cancer (4, 11.8%), and mental illness (3, 8.8%).

HIV status was reported in 20 of 34 studies (58.8%), with 10 including HIV-positive participants and 4 explicitly excluding them, while 14 studies did not report HIV status (Table 3; Fig 4). Drug resistance profiles were heterogeneous: drug‑susceptible studies (n = 14, 41.2%), drug‑resistant (n = 9, 26.5%), mixed (n = 9, 26.5%). Most studies investigated pulmonary TB (n = 25, 73.5%); a smaller proportion included both pulmonary and extrapulmonary disease (n = 7, 20.6%) or exclusively extrapulmonary cases (n = 2, 5.9%). Diabetes was the most reported non‑HIV comorbidity (n = 13, 38.2%).

**Table 3:** Population characteristics reported in included studies (N = 34).

Population Descriptor	Category	Studies, n (%)
**HIV status reporting**	HIV-positive participants included	10 (29.4)
	HIV-positive participants excluded	4 (11.8)
	HIV status reported but inclusion not specified	6 (17.6)
	HIV status not reported	14 (41.2)
**Drug resistance profile**	Drug-susceptible tuberculosis	14 (41.2)
	Drug-resistant tuberculosis	9 (26.5)
	Mixed resistance populations	9 (26.5)
	Not specified	2 (5.9)
**Tuberculosis disease site**	Pulmonary tuberculosis only	25 (73.5)
	Pulmonary and extrapulmonary tuberculosis	7 (20.6)
	Exclusively extrapulmonary tuberculosis	2 (5.9)
Non-HIV comorbidities reported*	Diabetes mellitus	13 (38.2)
	Liver disease	5 (14.7)
	Hypertension	4 (11.8)
	Cancer	4 (11.8)
	Mental illness	3 (8.8)

#### Outcome definitions.

Outcome definitions varied substantially across studies (Table 4). The most common definition was WHO-aligned bacteriological treatment failure (11 studies, 32.4%). Composite unfavourable outcomes (7 studies, 20.6%) and failure of sputum culture conversion (6 studies, 17.6%) were also frequent. Other definitions included emergence of drug resistance during treatment (2 studies), treatment non‑completion (2 studies), and other clinical endpoints (2 studies); four studies did not explicitly define the outcome.

**Table 4:** Operational definitions of treatment failure outcomes used in the included studies (N = 34).

Outcome definition category	Description	Studies, n (%)
WHO-aligned bacteriological failure	Persistent smear or culture positivity at ≥5 months or at treatment completion	11 (32.4)
Composite unfavorable outcome	Combined endpoints including treatment failure, death, loss to follow-up, or relapse	7 (20.6)
Failure of sputum culture conversion	Lack of sputum culture conversion at predefined time points (e.g., 2, 5, or 6 months)	6 (17.6)
Emergence of drug resistance during treatment	Development of new drug resistance during therapy	2 (5.9)
Treatment non-completion	Default, interruption, or abandonment of therapy	2 (5.9)
Other clinical endpoints	Disease-specific outcomes (e.g., inability to taper corticosteroids) or mortality	2 (5.9)
Outcome not explicitly defined	Studies predicting treatment failure without specifying diagnostic criteria	4 (11.8)

### Model characteristics

#### Machine learning algorithms and algorithm families.

A wide range of ML algorithms were evaluated. Among the 30 studies reporting a best-performing model, tree-based approaches were most frequently selected. Random forest was best in 9 studies (30.0%); decision trees/CART in 5 (16.7%); gradient boosting approaches (XGBoost, LightGBM) in 4 studies (13.3%); logistic regression models (including regularized variants)in 4 studies (13.3%), neural networks in 3 studies (10.0%); support vector machines in 2 studies (6.7%); and other approaches (k-nearest neighbors, elastic net models, and ensemble methods in 3 studies (10.0%).

When grouped into broader methodological families, tree-based methods dominated (18 studies, 52.9%), followed by regression-based models (logistic regression, LASSO, and elastic net) in 8 studies (23.5%), neural network/deep learning models in 4 studies (11.8%), and other ML approaches such as support vector machines and k-nearest neighbors in 4 studies (11.8%).

#### Data modalities and multimodal modeling.

Predictors were broadly grouped into several domains, including clinical and demographic characteristics (such as age, sex, symptoms, and medical history), laboratory biomarkers (blood tests and inflammatory markers), microbiological data (smear microscopy, culture, GeneXpert, and drug susceptibility testing), radiological or imaging features (e.g., chest X-ray or computed tomography), pharmacokinetic measures (such as drug concentrations and exposure metrics), genomic or other omics data, and social or behavioral factors including education, employment, income, alcohol use, smoking, and substance use [52,53].

**Fig 5:**
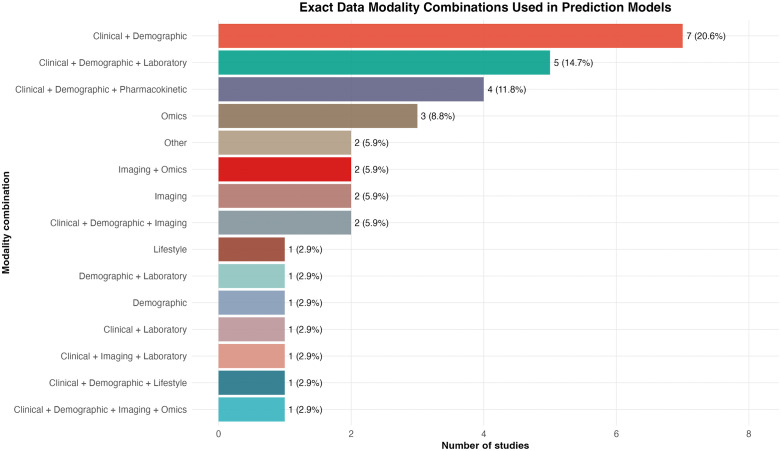
Data Modality Combinations Used in Prediction Models. Horizontal bar chart showing the specific combinations of data modalities used across the 34 included studies. The most frequent combinations were clinical plus demographic data only (7 studies, 20.6%), clinical plus demographic plus laboratory data (5, 14.7%), clinical plus demographic plus pharmacokinetic data (4, 11.8%), and omics data only (3, 8.8%). The variety of combinations reflects the diversity of approaches to multimodal modeling in tuberculosis treatment outcome prediction.

Most studies incorporated information from multiple domains rather than relying on a single type of data. Fig 5 illustrates the combinations used; the most frequent were clinical+demographic only (7 studies, 20.6%), clinical+demographic+laboratory (5, 14.7%), clinical + demographic + pharmacokinetic (4, 11.8%), and omics only (3, 8.8%)

**Fig 6:**
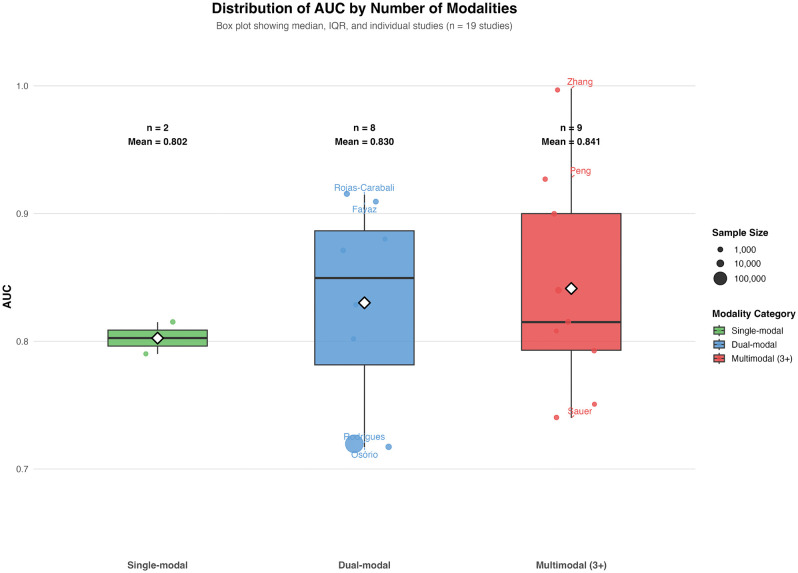
Distribution of Area Under the Curve by Number of Modalities. Box plot comparing area under the curve values across studies grouped by the number of data modalities used: single-modal (n = 2 studies), dual-modal (n = 8), and multimodal with three or more data types (n = 9). Boxes represent the interquartile range, horizontal lines indicate the median, and whiskers extend to the most extreme data points within 1.5 times the interquartile range. Individual study points are overlaid, with point size proportional to sample size. Mean area under the curve increased from 0.802 (single-modal) to 0.830 (dual-modal) to 0.841 (multimodal), but the overall difference was not statistically significant (Kruskal–Wallis p = 0.758). Selected studies are labeled for reference.

Model discrimination varied modestly according to the number of modalities. Among the 19 studies reporting AUC, single-modality models achieved a mean AUC of 0.802, dual-modality models achieved 0.830, and multimodal models integrating three or more data sources achieved 0.841 (Fig 6). The increase was not statistically significant (p = 0.758), suggesting data quality and relevance may be more important than quantity.

#### Feature selection, explainability, and tuning.

Feature selection was reported in 23 studies (67.6%) [43,54,55]. The most common approaches were filter methods (e.g., univariate statistical tests, mutual information, chi-square) in 9 studies, wrapper methods (recursive feature elimination/stepwise selection) in 7 studies, embedded methods (LASSO regularization/random forest importance) in 6 studies and Boruta algorithm in one study. Hyperparameter tuning was described in 20 studies (58.8%), most commonly grid search with cross-validation. Model explainability was reported in 12 studies (35.3%), most frequently through feature importance rankings, followed by SHAP values, decision tree visualizations, and odds ratios derived from logistic regression models.

### Predictive performance

#### Overall pooled discrimination.

**Fig 7:**
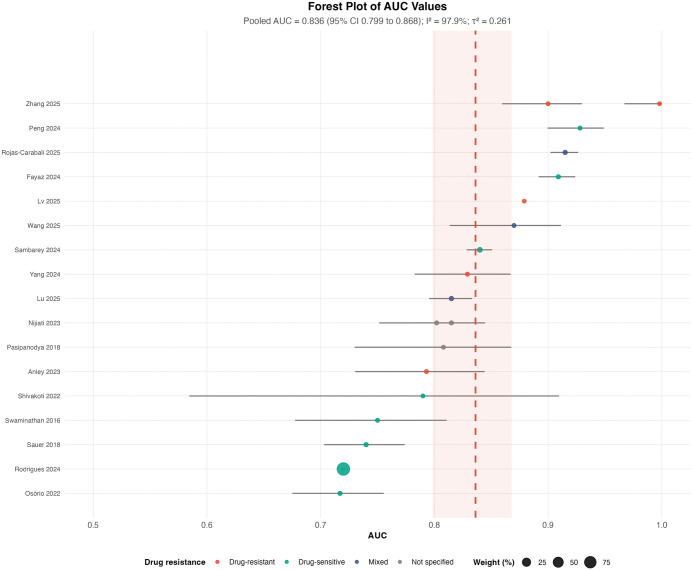
Forest Plot of Area Under the Curve Values. Forest plot displaying individual study area under the curve estimates with 95% confidence intervals for the 19 studies included in the meta-analysis. Squares represent individual study estimates, with square size proportional to the study’s weight in the random-effects meta-analysis. Horizontal lines indicate 95% confidence intervals. The vertical dashed line at area under the curve = 0.5 represents chance performance (no discrimination). The diamond at the bottom represents the pooled random-effects estimate of 0.836 (95% confidence interval 0.799–0.868). Substantial heterogeneity was observed (I² = 97.9%, τ² = 0.261, p < 0.001). Studies are labeled by first author and year; colors indicate drug resistance profile (drug-sensitive, mixed, not specified, or drug-resistant).

Nineteen studies (100,790 participants) contributed AUC data.. Study-level AUC ranged from 0.717 (95% CI 0.677–0.757) to 0.998 (95% CI not estimable). The pooled AUC was 0.836 (95% CI 0.799–0.868) (Fig 7).

Heterogeneity was extreme (I² = 97.9%, τ² = 0.261, Q p < 0.001). Variation in outcome definitions is a major contributor to this heterogeneity; therefore, the pooled estimate should be interpreted as a summary of central tendency rather than a generalisable performance benchmark.

#### Subgroup analyses.

**Fig 8:**
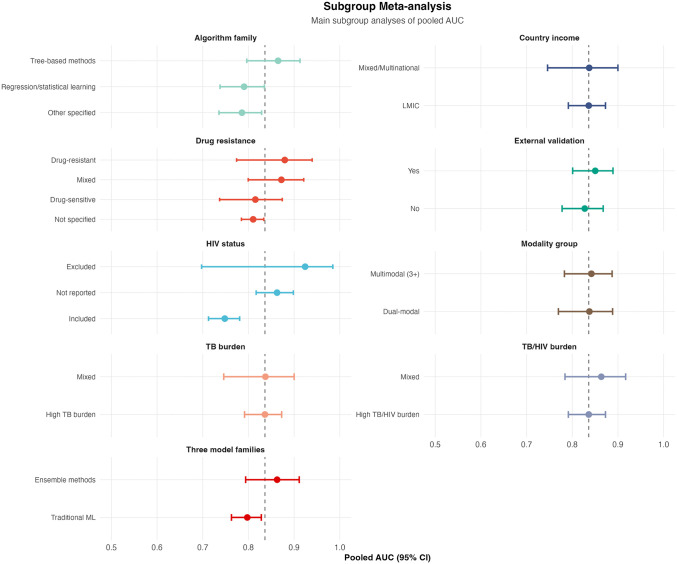
Subgroup Meta-Analysis of Pooled Area Under the Curve. Forest plot displaying pooled area under the curve estimates with 95% confidence intervals for all prespecified subgroup analyses. Subgroups are organized by category: drug resistance profile, HIV status, external validation, country income, tuberculosis burden, tuberculosis/HIV burden, algorithm family, broad algorithm family (traditional machine learning versus ensemble methods), and data modality group. The vertical dashed line at area under the curve = 0.836 indicates the overall pooled estimate. Notable findings include lower pooled area under the curve in studies including HIV-positive participants (0.748) compared to those excluding them (0.924), and higher performance in studies using clinical plus laboratory data (0.910) compared to clinical data alone (0.818).

Subgroup analyses (Table 5; Fig 8) showed that performance was higher in drug‑resistant tuberculosis (AUC 0.879) and mixed‑resistance populations (0.872) than in drug‑sensitive cohorts (0.815). Studies including HIV‑positive participants had substantially lower discrimination (0.748) compared with those that excluded them (0.924). Models with external validation performed similarly to those without (0.850 vs 0.827). By modelling approach, tree‑based/ensemble methods outperformed traditional statistical models (AUC ~ 0.86 vs ~ 0.80). Performance differences by data modality were modest; models incorporating laboratory data showed the highest AUC (~0.91). Across geographic and epidemiologic strata (LMIC, high TB burden, TB/HIV burden), pooled AUCs were similar (~0.836).

**Table 5:** Summary of subgroup meta-analysis findings.

Domain	Comparison	Studies (n)	Pooled AUC (95% CI)	I² (%)	Key Interpretation
Drug resistance	Drug-resistant	5	0.879 (0.774–0.940)	89.2	Higher signal in structured care settings
	Drug-sensitive	8	0.815 (0.737–0.874)	99.0	Greater heterogeneity
HIV status	Included	6	0.748 (0.713–0.781)	65.4	Reduced performance
	Excluded	4	0.924 (0.697–0.985)	96.9	Limited generalizability
External validation	Yes	6	0.850 (0.801–0.889)	85.7	Slightly higher performance
	No	13	0.827 (0.778–0.868)	97.8	Potential optimism bias
Algorithm class	Ensemble	9	0.863 (0.794–0.911)	99.1	Best overall performance
	Traditional ML	8	0.797 (0.763–0.828)	84.0	Lower discrimination
Data modality	Clinical + lab	4	0.910 (0.869–0.939)	63.0	Biomarker contribution
	Multimodal (3+)	9	0.842 (0.783–0.887)	96.2	No clear advantage
Geography	LMIC	15	0.836 (0.791–0.873)	96.7	Comparable performance

Abbreviations: AUC, area under the receiver operating characteristic curve; LMIC, low- and middle-income country; ML, machine learning.

Note: This table presents selected subgroup contrasts emphasized in the main text. Full subgroup results are shown in Fig 7 and supplementary subgroup outputs.

#### Meta-regression.

In univariable meta‑regression (Table 6), year of publication was positively associated with performance (β = 0.110, p = 0.010). Inclusion of HIV‑positive participants was associated with lower performance (β = −0.690, p = 0.002). Sample size, external validation, drug‑resistant focus, and high TB burden setting were not significantly associated with AUC. In the multivariable model, year remained independently associated (β = 0.125, p = 0.033), and sample size showed a borderline inverse association (β = −0.137, p = 0.080), suggesting smaller studies tended to report higher performance. The model explained approximately 43% of between‑study heterogeneity (R² = 43.0%), but substantial residual heterogeneity remained (I² = 90.3%, p < 0.001).

**Table 6:** Meta-regression of study-level factors associated with model performance. Associations with logit-transformed AUC.

Moderator	β coefficient	95% CI	P value	Note
**Multivariate**				
Year of publication (centered at 2015)	0.125	0.010 to 0.241	0.033	NA
Sample size (log-transformed)	−0.136	−0.289 to 0.016	0.080	NA
External validation	−0.161	−0.659 to 0.336	0.525	NA
HIV-positive participants included	−0.331	−0.923 to 0.262	0.274	NA
Drug-resistant tuberculosis focus	−0.034	−0.616 to 0.549	0.910	NA
**Univariate**				
Year of publication (centered at 2015)	0.110	0.026 to 0.194	0.010	NA
Sample size (log-transformed)	−0.068	−0.218 to 0.081	0.371	NA
External validation	0.255	−0.343 to 0.854	0.403	NA
HIV-positive participants included	−0.690	−1.135 to −0.245	0.002	NA
Drug-resistant tuberculosis focus	0.326	−0.339 to 0.991	0.337	NA
High TB burden setting	−0.007	−0.620 to 0.607	0.983	NA
LMIC setting	,	,	,	NA

**Abbreviations:** AUC, area under the receiver operating characteristic curve; LMIC, low- and middle-income country.

**Note:** Positive coefficients indicate higher logit-AUC with increasing values of the moderator. The multivariable model adjusted for year, sample size, external validation, HIV inclusion, and drug-resistant tuberculosis focus.

### Sensitivity analyses

Leave‑one‑out sensitivity analysis showed the pooled AUC remained stable (range 0.825–0.842). Restriction to studies with lower risk of bias (n = 4) yielded a pooled AUC of 0.851 (95% CI 0.778–0.909). Given the exploratory nature of multiple subgroup comparisons and the small sizes of some subgroups (e.g., imaging‑dominant, n = 2), these findings should be interpreted as hypothesis‑generating rather than confirmatory.

#### Predictor domains and knowledge gaps.

**Fig 9:**
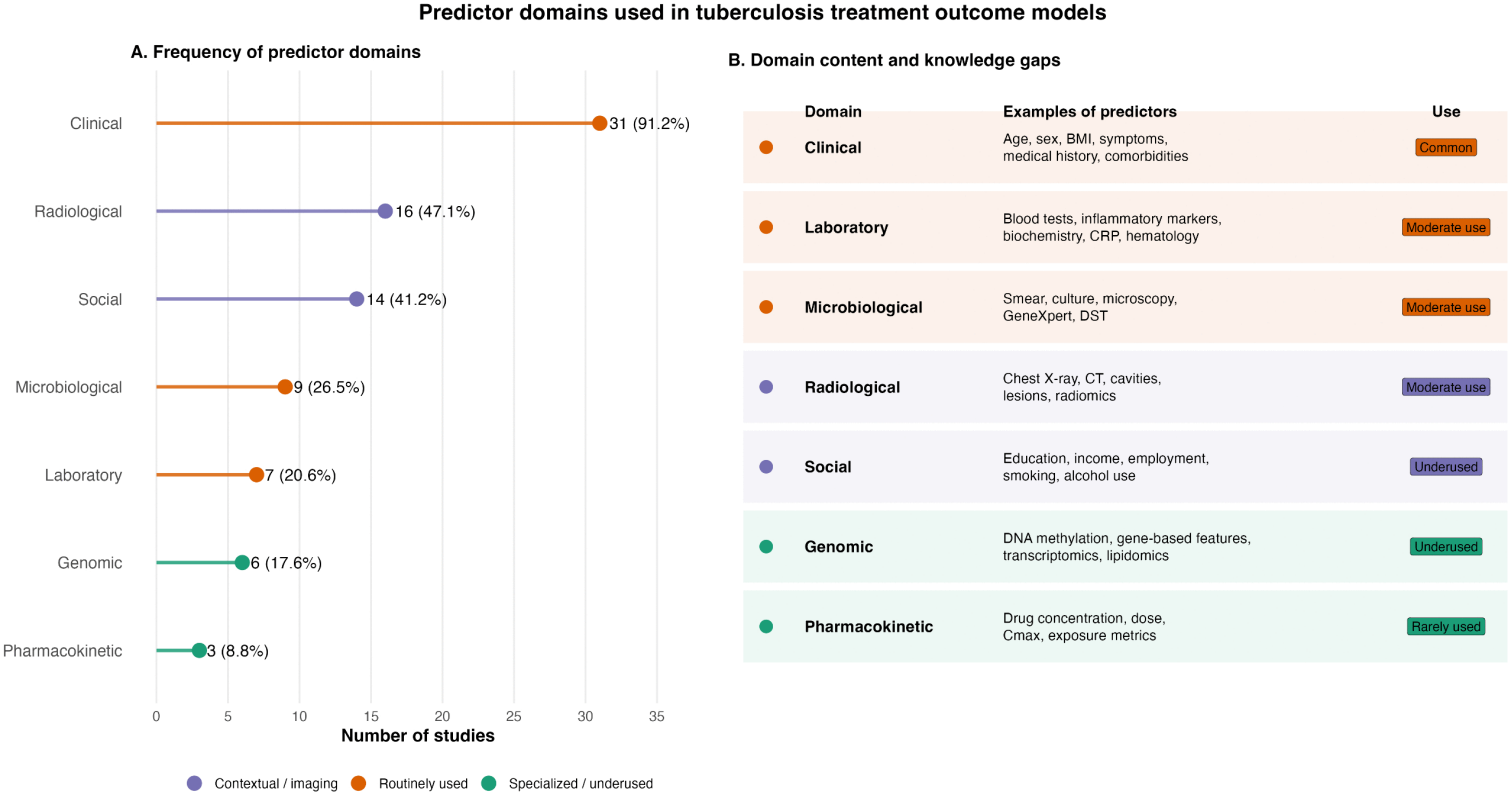
Predictor Domains Used in Tuberculosis Treatment Outcome Models. Two-panel figure summarizing predictor domains across the 34 included studies. Panel A: Frequency of Predictor Domains. Bar chart showing the number and percentage of studies using each predictor domain. Clinical and demographic variables were most common (31 studies, 91.2%), followed by social determinants (13, 38.2%) and radiological features (13, 38.2%). Microbiological data (9, 26.5%), laboratory biomarkers (7, 20.6%), genomic/omics data (4, 11.8%), and pharmacokinetic data (3, 8.8%) were used less frequently. Panel B: Domain Content and Knowledge Gaps. Table summarizing examples of predictors within each domain and their frequency of use. Social determinants, genomic/omics data, and pharmacokinetic data are identified as underused or rarely used, representing important knowledge gaps in the literature.

Clinical variables were used in 31 studies (91.2%); social factors and radiological features each in 13 (38.2%); microbiological in 9 (26.5%); laboratory markers in 7 (20.6%); genomic/omics in 4 (11.8%); and pharmacokinetic in 3 (8.8%) (Fig 9). Social determinants, pharmacokinetic data, and omics features were notably underused. Only two studies included paediatric populations, and no study focused exclusively on children or extrapulmonary disease.

**Methodological quality, validation rigor, and publication bias.**

#### Risk of bias.

**Fig 10:**
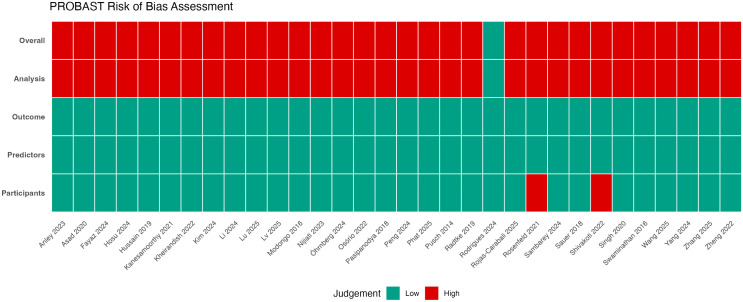
PROBAST Risk of Bias Assessment. Traffic light plot summarizing the risk of bias assessment using the Prediction model Risk Of Bias Assessment Tool (PROBAST). Each row represents one of the 34 included studies, and columns represent the four PROBAST domains (Participants, Predictors, Outcome, Analysis) plus the Overall rating. Green indicates low risk of bias, yellow indicates unclear risk, and red indicates high risk of bias. Only one study (2.9%) was rated as low risk of bias overall. The Analysis domain was the primary source of bias, with 33 studies (97.1%) rated as high risk. The Participants, Predictors, and Outcome domains were predominantly low risk.

Risk of bias was predominantly driven by deficiencies in the analysis domain (Fig 10; Table 7). The participants domain was low risk in 32 studies (94.1%), and predictors and outcome in all 34 (100%). However, the analysis domain was high risk in 33 studies (97.1%), primarily due to inadequate handling of missing data, lack of calibration assessment, and insufficient validation. Consequently, only one study (2.9%) was rated low risk overall

**Table 7:** Summary of risk of bias across PROBAST domains.

Summary of risk of bias across PROBAST domains			
Domain-level distribution of low, unclear, and high risk of bias judgments			
PROBAST domain	Low risk, n (%)	Unclear risk, n (%)	High risk, n (%)
Participants	32 (94.1)	0 (0.0)	2 (5.9)
Predictors	34 (100.0)	0 (0.0)	0 (0.0)
Outcome	34 (100.0)	0 (0.0)	0 (0.0)
Analysis	1 (2.9)	0 (0.0)	33 (97.1)
Overall	1 (2.9)	0 (0.0)	33 (97.1)

**Abbreviation:** PROBAST, Prediction model Risk Of Bias Assessment Tool.

**Note:** Percentages are calculated across the 34 included studies within each PROBAST domain.

#### Validation rigor.

Internal validation was reported in 30 studies (88.2%), predominantly using split‑sample or k‑fold cross‑validation. External validation was performed in only 8 studies (23.5%). Validation strategies varied in scope (geographic, temporal, cross‑cohort), but the small number of externally validated studies limits robust conclusions about generalizability.

#### Publication bias.

**Fig 11:**
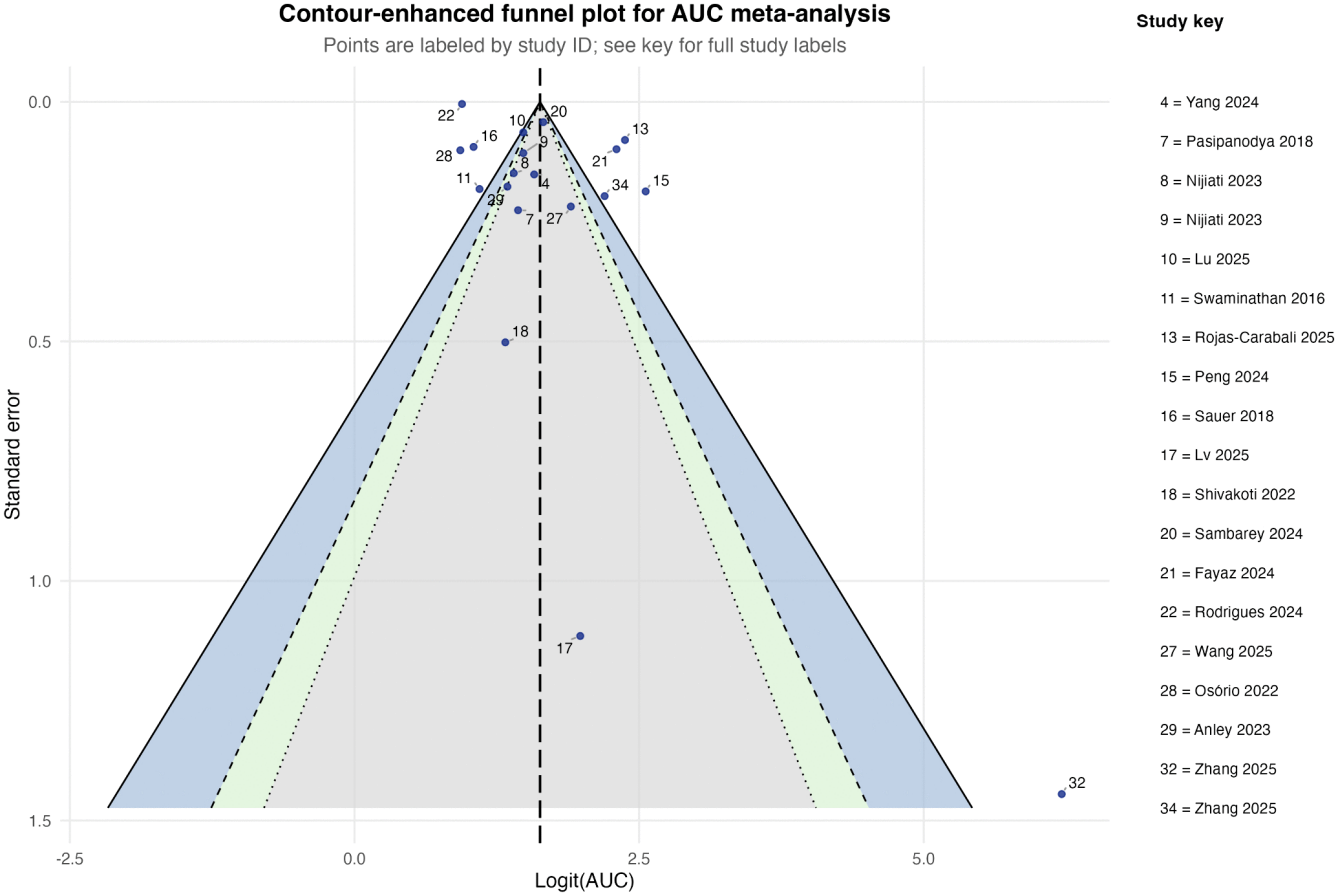
Contour-Enhanced Funnel Plot for Publication Bias Assessment. Contour-enhanced funnel plot for assessing publication bias among the 19 studies included in the area under the curve meta-analysis. Each point represents an individual study, plotted by its logit-transformed area under the curve (x-axis) against its standard error (y-axis). The vertical dashed line represents the pooled estimate. The triangular region represents the expected distribution of studies in the absence of publication bias or small-study effects. Colored contour regions (white, light gray, dark gray) indicate different levels of statistical significance for hypothetical missing studies. Points are labeled by study identification number; a key mapping identification numbers to author names and years is provided. Visual inspection suggests some asymmetry, with a relative absence of small studies (high standard error) reporting low area under the curve values. Egger’s test was significant (p = 0.024), indicating evidence of small-study effects or publication bias.

Publication bias was assessed using funnel plot asymmetry and Egger’s test (Fig 11). Funnel plot inspection suggested asymmetry (Fig 11), and Egger’s test was significant (p = 0.024). Trim‑and‑fill did not impute additional studies. The possibility of optimism bias, particularly in emerging computational fields, remains [56–58].

### Evidence gaps

Geographic representation was uneven; high‑burden regions in sub‑Saharan Africa and Southeast Asia were sparsely represented. HIV‑affected populations were under‑represented, and model performance was consistently lower in these groups. Social/behavioural predictors were included in fewer than 40% of studies. No studies focused exclusively on children or extrapulmonary tuberculosis. External validation was uncommon, calibration was rarely assessed, and no study evaluated real‑world implementation or health system variables.

## Discussion

This systematic review and meta-analysis of 34 studies evaluating AI/ML models for TB treatment failure prediction highlights a rapidly expanding field. While the pooled AUC of 0.836 suggests strong theoretical discriminative capacity, comparable to performance reported in other infectious disease prognostic models [14,59], this metric must be interpreted with extreme caution. The clinical utility of these models is currently severely constrained by extreme heterogeneity, a pervasive high risk of bias, and a lack of rigorous external validation.

A primary driver of the observed heterogeneity (I² = 97.9%) is the inconsistent definition of “treatment failure” across the primary literature. Included studies pooled highly varied endpoints, combining strictly defined WHO bacteriological failures with composite outcomes (including death or loss to follow-up) and interim culture conversion metrics. Because the biological and socio-structural drivers of these endpoints differ fundamentally, a unified interpretation of the pooled AUC is compromised. A model predicting loss to follow-up heavily weights social determinants, whereas one predicting bacteriological failure relies heavily on drug-resistance profiles.

Algorithmically, tree-based and ensemble methods outperformed traditional regression models. This aligns with broader ML literature [16,60], reflecting the capacity of ensemble models to map complex, non-linear interactions among clinical, demographic, and microbiological variables. However, we found that increasing the sheer volume of data modalities (multimodal modeling) did not yield statistically significant performance gains over simpler models. This suggests that the clinical relevance and quality of predictors (such as incorporating laboratory biomarkers) outweigh the mere quantity of data domains integrated [53].

Crucially, model performance dropped significantly when HIV-positive individuals were included in the training cohorts. TB-HIV co-infection introduces profound clinical complexity, altering immune responses, overlapping drug toxicities, and distinct disease presentations [61,62]. The underrepresentation of HIV-affected populations, coupled with an geographic imbalance that largely excludes high-burden sub-Saharan African settings [1,8], severely limits the generalizability and equity of current tools.

Furthermore, the PROBAST assessment revealed systemic methodological flaws. The near-universal failure to assess and report model calibration is a critical barrier to clinical translation [63,64]. A model with high discrimination may still output poorly calibrated, inaccurate risk probabilities, risking unsafe clinical decision-making. Coupled with statistical evidence of publication bias (Egger’s p = 0.024), it is highly likely that current literature overestimates true, real-world model performance.

To bridge the gap between computational development and clinical implementation, future research must shift from isolated model creation toward standardized methodology. Developers should adopt standardized WHO outcome definitions, strictly adhere to reporting guidelines such as TRIPOD, and prioritize rigorous external validation.

### Strengths and limitations

This study provides a comprehensive synthesis of AI/ML applications in TB prognosis, integrating a quantitative meta-analysis with a rigorous PROBAST risk-of-bias assessment. By exploring subgroup variations, we successfully identified critical performance disparities tied to HIV status and algorithm selection.

However, several limitations must be noted. First, the extreme statistical heterogeneity and inconsistent outcome definitions limit the robustness and generalizability of the pooled AUC. Second, the small number of studies within specific subgroups constrains the statistical power of our meta-regression analyses. Third, while our eligibility criteria included pediatric populations, the primary literature either focused exclusively on adults or failed to disaggregate pediatric data. Because pediatric TB differs fundamentally in pathophysiology, diagnosis, and treatment response compared to adult TB, this data gap masks critical age-related outcome heterogeneities. Finally, the overwhelming high risk of bias across the included studies means that the reported performance metrics likely suffer from optimism bias.

## Conclusion

Current AI/ML models for predicting tuberculosis treatment failure demonstrate high discriminative scores but remain strictly investigational. Their clinical readiness is critically undermined by systemic methodological flaws, lack of calibration reporting, and a heavy reliance on internal validation. Furthermore, the existing literature suffers from a severe representational bias, inadequately capturing pediatric populations, HIV-affected cohorts, and patients in high-burden, low-resource settings. Before these computational tools can be safely and equitably deployed in clinical care, future research must prioritize adherence to standardized reporting guidelines, the use of universal clinical outcome definitions, and rigorous external validation in the diverse programmatic environments where the TB burden is highest.

## Supporting information

S1 Fig PRISMA Flow Diagram. Flow diagram documenting the study selection process for the systematic review and meta-analysis. From an initial 1,672 records identified through database searching, 1,344 records were removed during initial filtering based on study focus (non-tuberculosis), language, non-article publication types, irrelevant categories, missing metadata, or retraction status. After removal of 11 duplicates, 317 abstracts were screened, of which 265 were excluded as not relevant (review studies, animal models, tuberculosis-focused but not prediction models). Fifty-two full-text articles were assessed for eligibility; 18 were excluded for using traditional statistical methods without a machine learning component or not focusing on treatment failure prediction. Thirty-four studies were included in the systematic review; 19 reported area under the curve values and were included in the meta-analysis.
(TIF)

S1 Table Study characteristics. (HTML)

S2 Table Probast study level. (HTML)

S4 File PROSPERO Registered Protocol. (PDF)

S5 File Search strategy. (DOCX)

S6 File PROSPERO cropped. (PDF)
