# Supplementary material for: Artificial Intelligence and Machine Learning-based prediction of tuberculosis treatment failure: A systematic review and meta-analysis
Source: PLoS One. 2026 Aug 17;21(8):e0355124. doi: 10.1371/journal.pone.0355124 (PMC13480653; doi:10.1371/journal.pone.0355124)
Supplement: Supplementary file 2 [file pone.0355124.s006.PDF]

Predicting Treatment Failure in Pulmonary Tuberculosis using Artificial Intelligence and Machine Learning: A Systematic Review and Meta-analysis

Rogers Kamulegeya, Derrick Semugenze, David Patrick Kateete, Rose Nabatanzi, Willy Ssengooba, Joyce Nakatumba, Florence Kivunike, Mercy Takuwa, Faridah Mugala, Andrew Katumba

Citation

Rogers Kamulegeya, Derrick Semugenze, David Patrick Kateete, Rose Nabatanzi, Willy Ssengooba, Joyce Nakatumba, Florence Kivunike, Mercy Takuwa, Faridah Mugala, Andrew Katumba. Predicting Treatment Failure in Pulmonary Tuberculosis using Artificial Intelligence and Machine Learning: A Systematic Review and Meta-analysis . PROSPERO 2025 CRD420251101443. Available from https://www.crd.york.ac.uk/PROSPERO/view/CRD420251101443.

REVIEW TITLE AND BASIC DETAILS

Review title

Predicting Treatment Failure in Pulmonary Tuberculosis using Artificial Intelligence and Machine Learning: A Systematic Review and Meta-analysis

Condition or domain being studied

Tuberculosis: Application of Machine learning models and Artificial Intelligence in predicting treatment failure in patients receiving treatment for active tuberculosis

Rationale for the review

Individual studies show the potential of AI/ML for predicting TB treatment failure, but there is significant heterogeneity in methods, populations, algorithms, and validation. A systematic synthesis is needed to summarize the overall performance of these models, identify key predictors, assess the methodological quality of existing studies, and highlight research gaps.

Review objectives

Summarize Overall Performance: Provide a comprehensive overview of how effectively AI/ML models predict TB treatment failure.

Identify Key Predictors: Determine the most influential variables used in these models

Assess Methodological Quality: Critically evaluate the quality and risk of bias in the primary studies using the PROBAST tool

Evaluate Validation Rigor: Assess the extent of model validation, especially external validation

Keywords
Tuberculosis; Treatment failure; Prognosis; Prediction; Artificial intelligence; Machine learning; Computational modelling; Systematic review and meta-analysis

Country
Uganda

ELIGIBILITY CRITERIA

Population

Included
Studies including patients (adults or children) diagnosed with any form of active tuberculosis (pulmonary or extrapulmonary, drug-sensitive or drug-resistant) undergoing pharmacological treatment

Excluded
Studies focused exclusively on latent TB infection (LTBI), TB diagnosis without subsequent treatment outcome analysis, or populations not undergoing standard pharmacological TB treatment regimens. Studies focused solely on transmission modeling without individual patient outcome prediction. Animal studies. Studies where TB is only one of multiple conditions analyzed without specific results reported for the TB cohort. Studies on patients with TB-HIV co-infection where outcomes are not stratified or analyzable specifically for TB treatment failure.

Intervention(s) or exposure(s)

Included
Studies developing, validating (internally or externally), or comparing AI or ML models for predicting TB treatment failure. [15, 33, 34] This includes supervised learning algorithms (e.g., logistic regression (when explicitly framed as ML), SVM, random forests, neural networks, gradient boosting), deep learning models, and potentially unsupervised learning approaches if applied to patient stratification predictive of outcome

Excluded
Studies using only traditional statistical methods (e.g., simple Cox regression, standard logistic regression not framed as an ML approach within the study) without incorporating AI/ML techniques. Studies describing only the theoretical framework of a model without application to patient data. Studies focused solely on image analysis (e.g., CXR, CT) for diagnosis or disease severity assessment unless the image features are explicitly used within a model to predict treatment failure. Review articles, editorials, commentaries, letters, case reports, conference

abstracts (unless full text provides sufficient detail for quality assessment and data extraction), protocols.

Comparator(s) or control(s)

This review does not have any comparators

Study design

Both randomized and nonrandomized study types will be included.

Context

Predicting treatment failure in patients receiving standard pharmacological TB treatment. Treatment failure is broadly defined, encompassing outcomes like microbiological failure, clinical failure, relapse, death, or loss to follow-up when defined as failure by the primary study.

TIMELINE OF THE REVIEW

Date of first submission to PROSPERO

24 July 2025

Review timeline

Start date: 10 July 2025. End date: 31 October 2025.

Date of registration in PROSPERO

11 December 2025

AVAILABILITY OF FULL PROTOCOL

Availability of full protocol

A full protocol has been written and uploaded to PROSPERO. The protocol will be made available after the review is completed.

SEARCHING AND SCREENING

Search for unpublished studies

Both published and unpublished studies will be sought.

Main bibliographic databases that will be searched

The main databases to be searched are *CENTRAL - Cochrane Central Register of Controlled Trials*, *CLIB - The Cochrane Library*, *Embase - Embase via Ovid*, *Embase.com*, *LILACS - Latin American and Caribbean Health Sciences Literature*, *MEDLINE*, *PubMed* and *Scopus*.

Search language restrictions

The review will only include studies published in English.

Search date restrictions

Databases will be searched for articles published from 1 January 2000, there are no search end date restrictions.

Other methods of identifying studies

No other methods will be used.

Link to search strategy

A full search strategy is available in the full protocol as described in the *Availability of full protocol* section

Selection process

Studies will be screened independently by at least two people (or person/machine combination) with a process to resolve differences.

Other relevant information about searching and screening

None

DATA COLLECTION PROCESS

Data extraction from published articles and reports

Data will be extracted independently by at least two people (or person/machine combination) with a process to resolve differences.

Authors will be asked to provide any required data not available in published reports.

Study risk of bias or quality assessment

Risk of bias will be assessed using: *PROBAST*

Data will be assessed independently by at least two people (or person/machine combination) with a process to resolve differences.

Additional information will **not** be sought from study investigators if required information is unclear or unavailable in the study publications/reports.

Reporting bias assessment

The review will assess reporting bias through visual (funnel plots) and statistical (Egger's test) methods if meta-analysis is feasible, use a broad search strategy to minimize publication bias, and evaluate selective reporting within studies using the PROBAST tool. All findings related to reporting bias will be transparently documented and discussed.

Certainty assessment

The overall certainty of the evidence will be evaluated through the PROBAST risk of bias assessment. The results will be discussed narratively, and studies at high risk of bias may be excluded from any meta-analysis.

OUTCOMES TO BE ANALYSED

Main outcomes

The primary outcomes are the predictive performance metrics of the AI/ML models, including the Area Under the Receiver Operating Characteristic Curve (AUC), accuracy, sensitivity, and specificity.

Additional outcomes

An additional key outcome is the identification and frequency analysis of predictors (features) used in the models to determine which variables are most commonly associated with treatment failure.

PLANNED DATA SYNTHESIS

Strategy for data synthesis

Narrative Synthesis: All included studies will be synthesized narratively, with results summarized in tables and text.

Meta-analysis: A quantitative meta-analysis of performance metrics (AUC, sensitivity, specificity) will be performed using a random-effects model if a subset of studies is sufficiently homogeneous. Heterogeneity will be explored via subgroup and meta-regression analyses.

CURRENT REVIEW STAGE

Stage of the review at this submission

Review stage	Started	Completed
Pilot work	✓	
Formal searching/study identification		
Screening search results against inclusion criteria		
Data extraction or receipt of IPD		
Risk of bias/quality assessment		
Data synthesis		

Review status

The review is currently planned or ongoing.

Publication of review results

Results of the review will be published in English.

REVIEW AFFILIATION, FUNDING AND PEER REVIEW

Review team members

Mr Rogers Kamulegeya (review guarantor and contact) Makerere University. Uganda.

No conflict of interest declared.

Mr Derrick Semugenze. Makerere University. Uganda.

No conflict of interest declared.

Associate Professor David Patrick Kateete. Makerere University. Uganda.

No conflict of interest declared.

Dr Rose Nabatanzi. Makerere University. Uganda.

No conflict of interest declared.

Dr Willy Ssengooba. Makerere University. Uganda.

No conflict of interest declared.

Dr Joyce Nakatumba. Makerere University. Uganda.

No conflict of interest declared.

Dr Florence Kivunike. Makerere University. Uganda.

No conflict of interest declared.

Ms Mercy Takuwa. Makerere University. Uganda.

No conflict of interest declared.

Ms Faridah Mugala. Makerere University. Uganda.

No conflict of interest declared.

Dr Andrew Katumba. Makerere University. Uganda.

No conflict of interest declared.

Named contact
Mr Rogers Kamulegeya (vicrogerbre@gmail.com). Makerere University. Uganda.

Review affiliation
Makerere University

Funding source
Review has no specific/external funding but is supported by guarantor/review team (non-commercial) institutions.

Peer review
There has been no peer review of this planned review.

ADDITIONAL INFORMATION

Review conflict of interest
Declared individual interests are recorded under team member details.. No additional interests are recorded for this review.

Medical Subject Headings
Tuberculosis; Tuberculosis, Pulmonary; Treatment Failure; Artificial Intelligence; Machine Learning; Prognosis; Predictive Value of Tests; Recurrence; Mycobacterium; Tuberculosis, Multidrug-Resistant; Extensively Drug-Resistant Tuberculosis; Neural Networks, Computer; Support Vector

Machine; Patient-Specific Modeling; Deep Learning; Decision Trees; Algorithms; Mortality; Risk Factors; Treatment Outcome

SIMILAR REVIEWS

Check for similar records already in PROSPERO

PROSPERO identified a number of existing PROSPERO records that were similar to this one (last check made on 10 July 2025). These are shown below along with the reasons given by that the review team for the reviews being different and/or proceeding.

- Machine Learning-Based Risk Stratification for Postoperative Nausea and Vomiting: A Systematic Review of AI-Driven Predictive Models Since 2020. [published 6 March 2025] [CRD420251005955]. The review was judged **not to be similar**
- Machine Learning for the Diagnosis of Myocarditis: A Meta-Analytic Review of Model Performance [published 18 April 2025] [CRD420251035434]. The review was judged **not to be similar**
- Predictive performance of machine learning algorithms for multidrug-resistant tuberculosis: a systematic review and meta-analysis [published 4 July 2025] [CRD420251086624]. The review was judged **not to be similar**

PROSPERO version history

- Version 1.0, published 11 Dec 2025

Disclaimer

The content of this record displays the information provided by the review team. PROSPERO does not peer review registration records or endorse their content.

PROSPERO accepts and posts the information provided in good faith; responsibility for record content rests with the review team. The guarantor for this record has affirmed that the information provided is truthful and that they understand that deliberate provision of inaccurate information may be construed as scientific misconduct.

PROSPERO does not accept any liability for the content provided in this record or for its use. Readers use the information provided in this record at their own risk.

Any enquiries about the record should be referred to the named review contact
